# Effect of a Web-Based Intervention to Promote Physical Activity and Improve Health Among Physically Inactive Adults: A Population-Based Randomized Controlled Trial

**DOI:** 10.2196/jmir.2109

**Published:** 2012-10-30

**Authors:** Andreas Wolff Hansen, Morten Grønbæk, Jørn Wulff Helge, Maria Severin, Tine Curtis, Janne Schurmann Tolstrup

**Affiliations:** ^1^National Institute of Public HealthUniversity of Southern DenmarkCopenhagenDenmark; ^2^Centre of Healthy AgingDepartment of Biomedical SciencesUniversity of CopenhagenCopenhagenDenmark

**Keywords:** Intervention study: computer intervention, health behavior, primary prevention, adults

## Abstract

**Background:**

Many people in Western countries do not follow public health physical activity (PA) recommendations. Web-based interventions provide cost- and time-efficient means of delivering individually targeted lifestyle modification at a population level.

**Objective:**

To examine whether access to a website with individually tailored feedback and suggestions on how to increase PA led to improved PA, anthropometrics, and health measurements.

**Methods:**

Physically inactive adults (n = 12,287) participating in a nationwide eHealth survey and health examination in Denmark were randomly assigned to either an intervention (website) (n = 6055) or a no-intervention control group (n = 6232) in 2008. The intervention website was founded on the theories of stages of change and of planned behavior and, apart from a forum page where a physiotherapist answered questions about PA and training, was fully automated. After 3 and again after 6 months we emailed participants invitations to answer a Web-based follow-up questionnaire, which included the long version of the International Physical Activity Questionnaire. A subgroup of participants (n = 1190) were invited to a follow-up health examination at 3 months.

**Results:**

Less than 22.0% (694/3156) of the participants logged on to the website once and only 7.0% (222/3159) logged on frequently. We found no difference in PA level between the website and control groups at 3- and 6-month follow-ups. By dividing participants into three groups according to use of the intervention website, we found a significant difference in total and leisure-time PA in the website group. The follow-up health examination showed no significant reductions in body mass index, waist circumference, body fat percentage, and blood pressure, or improvements in arm strength and aerobic fitness in the website group.

**Conclusions:**

Based on our findings, we suggest that active users of a Web-based PA intervention can improve their level of PA. However, for unmotivated users, single-tailored feedback may be too brief. Future research should focus on developing more sophisticated interventions with the potential to reach both motivated and unmotivated sedentary individuals.

**Trial Registration:**

Clinicaltrials.gov NCT01295203; http://clinicaltrials.gov/ct2/show/NCT01295203 (Archived by WebCite at http://www.webcitation.org/6B7HDMqiQ)

## Introduction

Physical activity (PA) is associated with lower mortality and morbidity rates from cardiovascular disease, diabetes mellitus, cancer, and osteoporosis [1-6]. Despite the health benefits of PA, many people in Western countries do not follow public health PA recommendations [7,8].

Web-based interventions have been successfully applied to improve lifestyle and change health behavior targeting weight loss, stress management, fall-related injuries, smoking cessation, and heavy drinking [9-12]. The Internet has several advantages in delivering health promotion—for example, cost and time effectiveness, 24-hour accessibility, and generation of instant personalized or individually tailored feedback [13-15]. Individually tailored feedback, rather than a more general prevention message, is likely to be more effective, as users can identify with relevant personal information rather than general information [15-19]. Overall, Web-based interventions provide cost- and time-efficient means of delivering individually targeted lifestyle modification at a population level. Based on the existing Web-based randomized controlled trials in relation to PA, it is unclear whether the Internet can effectively deliver PA interventions [20-22]. A review from 2009 [23] identified Web-based PA interventions in primary prevention; only 4 of 16 studies had above-average external validity and reported a positive between-group effect in PA. Basically, two approaches exist in PA Web-based interventions. PA is either the only target in the intervention or included in a general lifestyle intervention targeting several behaviors simultaneously. However, evidence supporting the one or the other is inconclusive [22,23]. Many Web-based interventions conducted to date have limitations. Some studies used an inadequately powered sample, lacked a no-information control group, had a large dropout rate, or relied only on self-reported outcome measures.

The aim of this study was to examine whether an automated Web-based intervention would lead to increased PA among inactive persons in a large population. More specifically, we aimed to determine whether access to a website with individually tailored feedback on PA level and suggestions to increase PA would result in improvements in self-reported PA, anthropometrics, and physiological measurements in an intervention group compared with a no-information control group.

## Methods

### Setting

The intervention study was nested in the Danish Health Examination Survey 2007-2008 (DANHES) [24], a nationwide health study in Denmark. DANHES was carried out in 13 municipalities in 2007-2008 and comprised a Web-based questionnaire and a health examination test. All adults (18+ years of age) in 13 municipalities (N = 538,497) were invited by letter to take part in a Web-based questionnaire. The questionnaire assessed lifestyle-related health aspects with a focus on diet, smoking, alcohol use, and exercise. A representative sample in each of the 13 municipalities were also invited to a health examination test (n = 180,103). For details see Eriksen et al [24].

### Recruitment of Participants

The DANHES was used to recruit participants and as a baseline assessment. The intervention study was conducted in 11 of the 13 municipalities participating in DANHES during May 2008–May 2009. We excluded 2 municipalities from the intervention study, as one served as a pilot study for DANHES and paper questionnaires were used in the other.

The main inclusion criterion for participating in the intervention study was being physically inactive during leisure time. This was defined by the participants’ answer to a 4-category question describing PA level in leisure time. We included participants in the lowest 2 categories, mostly sedentary or light activities, in the study and excluded participants in the highest categories, moderate and vigorous PA. Further exclusion criteria were presence of serious heart problems, not being able to perform everyday activities, or missing values in the International Physical Activity Questionnaire (IPAQ) and the leisure-time PA question.

We identified participants who met the inclusion criteria by a screening program and invited them to join the intervention study at the end of the questionnaire in DANHES. If willing to participate, each participant was randomly assigned by the registration program to either an intervention (website) or a no-intervention control group. The only incentive given to participants was the possibility of being assigned to the intervention group. Blinding was not feasible.

The participants in the website group received an email with a link to a PA website immediately after allocation to the website group. In addition, relevant data from the health survey were automatically transferred to the intervention website. To access the website, the participants were required to log on to the website, using the same personal username and password given in the health survey.

All participants gave informed consent before being enrolled in the study. The study was approved by the Danish National Committee on Biomedical Research Ethics (H-D-2008-035).

### The Intervention Website

The intervention website was founded on the theories of stages of change [25] and of planned behavior [26]. First, we identified determinants in the theory: intentions, attitudes, self-efficacy, skills, social support, and knowledge. Second, we specified key objectives for each determinant and decided where in the intervention the objectives would be implemented (Table 1). The key objectives were used in the wider planning of the intervention to specify how objectives could be translated into action in the real-life intervention.

**Table 1:** Key determinants and objectives, and how they were incorporated in the Web-based physical activity (PA) intervention.

Determinant	Objective	Used in the intervention
Intentions	Have intention to increase PA	Tailored feedback or emails sent to users
	Maintain motivation and intention	Personal data (biofeedback)
Attitudes	Experience that PA is important and requires an extraordinary effort	Tailored feedback or emails sent to users
Self-efficacy	Be confident that PA can be increased	Tailored feedback or emails sent to users
Skills	Demonstrate skills to set goals	Goal setting
	Can identify situations where the new behavior is being challenged	General recommendations
Social and external support	Know that help can be provided by an expert	The forum for users
	Know that equals can be found in the forum	The forum for users
	Receive positive feedback from other users of the forum	The forum for users
	Know that family and friends in the near social environment can support and participate in the new behavior	General recommendations
Knowledge	Gain knowledge of how PA can be increased and which kind of activities are best suited	Tailored feedback, general training programs
	Know how increased PA can benefit everyday life	Tailored feedback, general recommendations

The website was structured as three major parts: (1) a personal page, which included individually tailored PA advice and a personal profile, (2) a page with training programs and general recommendations, and (3) a forum and discussion page for questions from participants.

The individually tailored PA advice consisted of three parts: (1) a general introduction, (2) normative feedback, which related the participant’s PA to the current PA recommendations and (3) general advice about using the tools on the website. The normative feedback was based on the summarized PA time from the participant’s answers in the IPAQ. Feedback was given in the domains of everyday activity, fitness training, and strength training. In each domain we defined categories in which the participants received tailored feedback according to their level of PA. The categories were partly based on PA recommendations from the Danish National Board of Health translated into minutes per week [27], together with analyses from answers in the IPAQ from 2 municipalities. The analyses showed that the categories needed to be wide because of overreporting in the IPAQ. The categories were everyday activity, being the total activity time from questions in the IPAQ summarized into low (<1200 min/week), moderate (1200-3500 min/week), and high levels (>3500 min/week); fitness training, being summarized time from vigorous and moderate-intensity activity from the transport and the leisure-time domain and moderate-intensity activity from the domestic domain in the IPAQ, at low (<40 min/week), moderate (40-350 min/week), and high (>350 min/week) levels; and strength training, being summarized time from the highest intensity level in the 4 domains in the IPAQ, at low (<100 min/week) and moderate to high (>100 min/week) levels. Participants who were 60 years of age or older were given extra advice regarding the importance of strength training. To monitor their progress during the study, the participants could register personal data, such as waist circumference and the result of a short fitness and strength test in the personal profile. We used this kind of biofeedback to keep the participants motivated during the intervention. Furthermore, current activity per day could be calculated with a short activity calculator, and it was possible to set goals for the following 4 weeks. We decided on 4 weeks for goal setting as a balance between time for seeing results and maintaining motivation. All participants were encouraged to make a personal profile to set their goals, monitor their progress, and implement their goals.

The part of the website that included the page with the training programs and general recommendations was structured in the same way as the PA advice in the 3 domains: everyday activity, fitness training, and strength training. The participants were encouraged to go through the different suggestions and programs and pick the ones that suited them best based on the individually tailored advice and goals set by each participant. General information about motivation and relevant links were also presented on the website.

On the forum and discussion page, a physiotherapist experienced in PA counseling answered all questions about PA and training from participants. In addition, participants could share experiences and give each other tips or search for training partners in a second forum.

We kept the tailored PA advice short so as not to overload the participant with information and, apart from training programs and general recommendations, as a means of following the theory of stages of change [25]. The theory describes how change is a process of progressing through a series of stages: precontemplation, contemplation, preparation, action, maintenance, and termination. Hence, participants ready for change and already in the action stage could make a personal profile, set goals, and find training programs. Participants in the precontemplation to preparation stage who did not log on to the website or did not make a personal profile were sent two email reminders to encourage them to become involved with the intervention. Participants in the maintenance stage who made a personal profile were sent reminders and encouraging emails to keep the profile updated after 4, 8, 12, and 16 weeks.

The content of the website was developed by the research team. Two professional Web companies did the graphic design and implementation. The intervention was pretested among experts and representatives of the target population. We tested screening of participants, invitation to the intervention, automatic generation of individually tailored advice, email generation, and general usability of the website. Furthermore, we used the first municipality participating in the study (n = 1298) for a pilot study. Comments and suggestions from these participants were used to fine-tune the website. Multimedia Appendix 1 shows recruitment of participants and the intervention website.

### Baseline and Follow-Up Health Examination

The baseline health examination included measurements of blood pressure, height, weight, body fat percentage, and grip strength. Aerobic fitness was estimated from a watt-max [28] or a sub-max test [29] on an ergometer bike. Body mass index (BMI) was calculated from measurements of height and body weight. For further details about methods and test procedures, see Eriksen et al [24].

From 3 selected municipalities in the intervention study, we invited 1190 participants to a follow-up health examination after 12 weeks. The participants were invited by email, which was sent 3 to 4 weeks prior to the examination. If the participant did not respond, a reminder email was sent 1 week after the first. The follow-up examination included the same measurements as the baseline health examination, and the same test procedures were followed. The follow-up examination was blinded to the examiners. After the health examination, the participants received their results to track developments from baseline to follow-up. We expected to see improvements in measurements included in the health examination, as participants in the intervention group were encouraged to engage in moderate- and high-intensity PA on a weekly basis. Improvements have been seen after 3 months in other studies depending on diet [30-32].

### Baseline and Follow-Up Questionnaire

The baseline questionnaire included demographics, health, lifestyle, and health behavior. We used the long version of the IPAQ, which is known to be a valid and reliable instrument for assessing PA [33], both at baseline and at follow-up. It consists of 31 items that collect information on PA in the 4 domains work, transport, housework and gardening, and leisure time. Motivation for changing PA behavior was assessed by the question “Do you wish to be more physically active than you already are?” (yes, yes/maybe, or no).

After 3 months and after 6 months, we invited all participants by email to answer a follow-up questionnaire. The follow-up questionnaire included questions about use of the website for the website group. Due to a technical error, only half the participants were invited to answer the 3-month follow-up questionnaire.

### Outcome Measures

The primary analysis in this intervention was overall level of PA based on self-reported PA from the IPAQ. The secondary outcome measures were blood pressure, height, weight, body fat percentage, and grip strength. The outcome measures were specified a priori.

As a post hoc outcome measure, a secondary analysis was carried out among active users of the intervention. Here we divided the participants of the website group into three groups according to user activity (no log-on, log-on once, and log-on more than once) and assessed level of PA. Furthermore, we calculated the odds ratios of being an active user of the website.

Power Estimates

We assumed that a reasonable effect of the intervention on total PA time estimated by the IPAQ would be around 12% for the intervention group and 5% for the control group. With a power of 80% probability of detecting a 12% versus 5% difference as statistically significant at the 5% level, we calculated the minimum sample size to be 250 in each group. We expected that approximately 50% of the participants in DANHES were sedentary. Assuming that 80% accepted participation and 25% were lost to follow-up, this would still give us a large population and hence ensure sufficient power.

### Statistical Analyses

We analyzed the IPAQ results according to the *Guidelines for Data Processing and Analysis of the International Physical Activity Questionnaire* [34] with the exception that we included participants with a missing value in day or time in the follow-up analysis. Nonparametric analyses were used, since IPAQ data were not distributed normally (Wilcoxon rank sum test between two groups and Kruskal-Wallis test between more than two groups).

Results were primarily analyzed as intention-to-treat analyses with the use of the last observation carried forward to account for missing data at follow-up. We analyzed completer data including only participants who completed the follow-up health examination or questionnaire.

Website use was assessed by the follow-up questionnaire and combined with information provided by the company that was responsible for the website, which recorded whether a participant logged on.

Odds ratios of being an active user (several log-ons versus one or none) were calculated in relation to sex, highest education level (<10, 10–12, 13–14, or 15+ years), age group (18–44, 45–64, or 65+ years), and motivation to be more active (yes, yes/maybe, no) by the use of logistic regression.

For all statistical calculations and analyses, we used Stata version 11.2 (StataCorp LP, College Station, TX, USA). We performed chi-square tests to examine differences in proportions between the groups. We considered *P*< .05 as statistically significant.

## Results

### Participation

**Figure 1:**
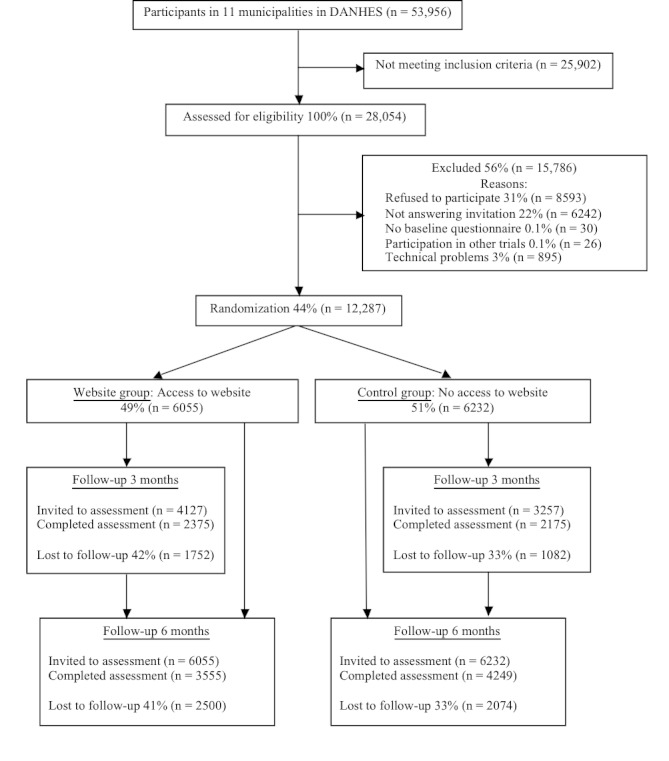
Flow diagram of the Web-based intervention (Denmark, 2008). DANHES = Danish Health Examination Survey 2007–2008.

In total, 53,956 persons participated in DANHES in the 11 municipalities. Of these, 28,054 participants met the inclusion criterion and were invited to participate in the intervention study (Figure 1). Among these, reasons for nonparticipation were refusal (8593) and not answering the invitation (6242). A technical error gave some participants in the control group access to the website and resulted in exclusion of 895 participants. A total of 12,287 participants were enrolled in the study, resulting in a 43.80% participation rate. The response rates in the 3-month questionnaire were 57.55% (2375/4127) in the intervention group and 66.41% (2175/3257) in the website group.

In the health examination, 32 participants were excluded due to participation in another health intervention, leaving 583 in the website group and 585 in the control group. In total, 434 (37.2%) participated in the follow-up health examination, with 215 in the intervention groupand 219 in the control group. Participants who were lost to follow-up in the health examination and questionnaire were not significantly different from those who completed follow-up in respect to baseline characteristics (data not shown).

### Baseline

Baseline characteristics did not differ significantly between the website and control groups as shown in Table 2. Mean age of the participants was 50 (SD 13.6) years and 64.82% (3925/6055) were women; overall, 18.95% (2329/12,287) were mostly sedentary and the rest reported light PA in their leisure time.

No significant differences were found in baseline characteristics between the website and control groups in the health examination (Table 3). The mean BMI among participants was 25 (SD 3.8) kg/m^2^and mean body fat percentage was 30.5% (SD 8.1). Aerobic fitness was somewhat low at 32 (SD 7.8) mL/min/kg.

### Follow-Up

Participants in the website group did not report a significantly different level of PA compared with the control group at the 6-month follow-up (Table 4).

The result was the same in the completer analysis and analysis at 3-month follow-up (data not shown). Analyzing participants who stated that they wanted to change their PA separately gave the same result (data not shown). In relation to other health and baseline characteristics, no significant differences were found at follow-up at 3 and 6 months (data not shown). Furthermore, no significant changes were found in either the website or the control group from baseline to follow-up (data not shown).

The results from the follow-up health examination showed no significant differences between the website and control groups (Table 5). From baseline to follow-up, we found no significant changes in either the website or the control group (data not shown).

**Figure 2:**
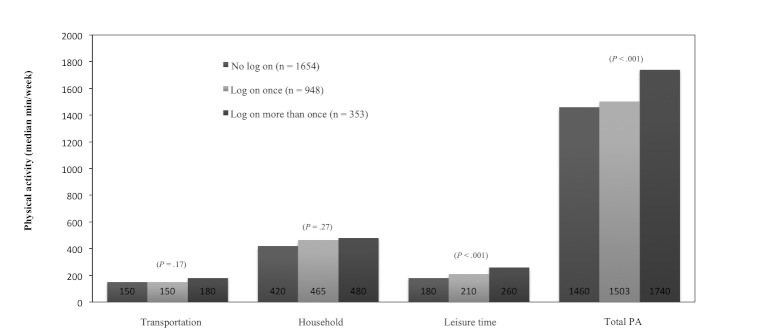
Physical activity (PA) in three domains and total PA at 6 months by frequency of log-ons to intervention website in the website group. Kruskal-Wallis test for differences between the groups (Denmark, 2008).

When we divided the participants into three groups according to use of the intervention website (no log-on, log-on once, and log-on more than once), we found a significant difference between the groups in leisure-time PA and total PA (Figure 2). Dividing participants from the website group who participated in the follow-up health examination into the same three subgroups of website use did not show significant differences between the groups (data not shown).

**Table 2:** Baseline characteristics of the participants by website and control group (Denmark, 2008).

Characteristic		Website group (n = 6055)		Control group (n = 6232)		*P* value^a^
Age (years), mean (SD)		50.7	(13.6)	50.4	(13.7)	.31
Sex (women), n (%)		3924	(64.8%)	4043	(64.9%)	.96
**Education (years), n (%)**						.18
	<10	461	(7.6%)	430	(6.9%)	
	10–12	1212	(20.02%)	1259	(20.20%)	
	12–14	1491	(24.62%)	1470	(23.59%)	
	15+	2891	(47.75%)	3073	(49.31%)	
**Physical activity in leisure time, n (%)**						.55
	Sedentary	1157	(19.11%)	1172	(18.81%)	
	Low	4898	(80.89%)	5060	(81.19%)	
	Vigorous or moderate	0	0	0	0	
**Physical activity by IPAQ** ^b^ **(min/week), median (25th–75th percentile)** ^c^						
	Work	60	(0–780)	60	(0–780)	.66
	Transportation	160	(40–390)	180	(45–390)	.37
	Household	500	(180–1110)	480	(180–1080)	.04
	Leisure time	200	(60–465)	195	(60–420)	.15
	Sitting	2310	(1650–3180)	2340	(1680–3300)	.06
	Total PA	1600	(840–2640)	1560	(840–2485)	.11
Wish to be more physically active (yes), n (%)		3197	(52.80%)	3372	(54.11%)	.32
Self-rated health good or very good, n (%)		4323	(71.40%)	4482	(71.92%)	.53

^a^ Independent *t* test for difference between groups for age and chi-square test for differences between groups in categorical characteristics.

^b^International Physical Activity Questionnaire.

^c^ Website group n = 4435 and control group n = 4509. Wilcoxon rank sum test for difference between groups.

**Table 3:** Baseline characteristics of the subsample of participants in the health examination by website and control group (Denmark, 2008).

Characteristic	Website group (n = 583)		Control group (n = 585)		*P* value^a^
Sex (women), n (%)	345	(59.2%)	332	(56.8%)	
Age (years), mean (SD)	51.2	(13.9)	50.5	(13.2)	.39
BMI^b^ (kg/m^2^), mean (SD)	25.4	(3.8)	25.0	(3.8)	.12
Waist circumference (cm), mean (SD)	90.1	(12.0)	89.6	(11.8)	.43
Body fat (%), mean (SD)	30.4	(8.2)	30.5	(8.0)	.87
Systolic blood pressure (mmHg), mean (SD)	125.0	(16.8)	123.1	(16.3)	.05
Diastolic blood pressure (mmHg), mean (SD)	79.7	(10.0)	78.9	(10.6)	.15
Arm strength (kg), mean (SD)	26.9	(9.4)	29.3	(9.6)	.37
Aerobic fitness^c^ (mL/min/kg), mean (SD)	32.0	(7.9)	31.5	(7.7)	.85

^a^ Independent *t* test for difference between groups and chi-square test for differences between groups in categorical characteristics.

^b^ Body mass index.

^c^ Aerobic fitness total either from watt-max or 1-point test.

**Table 4:** Physical activity assessed by International Physical Activity Questionnaire (min/week) at 6-month follow-up by website and control group (intention-to-treat analysis) (Denmark, 2008).

Type of physical activity^a^	Website group: (n = 4435)		Control group (n = 4509)		*P* value^b^
Work	60	(0–800)	60	(0–825)	.62
Transportation	180	(45–400)	200	(60–420)	.62
Household	480	(180–1080)	480	(180–1080)	.17
Leisure time	200	(60–450)	200	(60–420)	.25
Sitting	2220	(1500–3060)	2220	(1500–3150)	.52
Total physical activity	1575	(845–2580)	1560	(840–2520)	.25

^a^ Variables are shown as median (25th–75th percentile).

^b^ Wilcoxon rank sum for difference between groups.

**Table 5:** Health examination measurements of the subsample of participants at 3-month follow-up by website and control group (intention-to-treat analysis) (Denmark, 2008).

Measurement^a^	Website group (n = 583)		Control group (n = 585)		*P* value^b^
BMI^c^ (kg/m^2^)	25.3	(0.2)	25.0	(0.2)	.12
Waist circumference (cm)	90.0	(0.5)	89.1	(0.5)	.34
Body fat (%)	30.4	(0.3)	30.5	(0.3)	.87
Systolic blood pressure (mmHg)	125.1	(0.7)	123.1	(0.7)	.04
Diastolic blood pressure (mmHg)	79.4	(0.4)	78.5	(0.4)	.12
Arm strength (kg)	27.5	(0.5)	26.9	(0.4)	.32
Aerobic fitness^d^ (mL/min/kg)	31.6	(0.4)	31.8	(0.3)	.70

^a^ Variables are shown as mean (SE).

^b^ Independent *t* test for difference between groups.

^c^ Body mass index.

^d^ Aerobic fitness total either from watt-max or 1-point test.

### Website Users

We found that 22.0% (694/3156) of the website group logged on to the website once and only 7.0% (222/3159) logged on more than once (Table 6). The question about website use was answered by 3245 participants, of whom 73 (2%) reported that they had logged on to the website, even though reports from the website company responsible for the website showed that they did not log on to the website. We excluded these participants in the analyses of website users. According to 70.0% (710/1014) of the respondents in the website group, forgetfulness was the main reason for not logging on to the website (data not shown).

In the group of participants who did log on to the website, 31.4% (318/1014) believed that the intervention helped them to increase PA. When we analyzed this group separately, we did not find significant changes in PA from baseline to follow-up (data not shown). The website’s PA adviser received few questions and the participant forum was not used at all.

Analysis of the active users of the intervention in relation to age group, educational level, motivation, and sex showed that participants in the age group 45–64 years (odds ratio 1.6, 95% confidence interval 1.2–2.1) and 65+ years (odds ratio 2.0, 95% confidence interval 1.4–2.8) were more likely to log on more than once than were those in the age group 18–44 years. Motivated participants (odds ratio 1.5, 95% confidence interval 1.0–2.1) were more likely than nonmotivated participants to log on more than once.

**Table 6:** Use of the intervention website at 6-month follow-up in the website group (Denmark, 2008)^a^.

Website use		n	%
**How often did you use the website during the last 6 months?** (n = 3159)			
	I have not logged on to the website	2243	71.00%
	I have logged on to the website once	694	22.0%
	I have logged on to the website several times	159	5.0%
	I have logged on to the website several times and made a personal profile	63	2%
**Has the website helped you to increase your physical activity level? (n = 1014)**			
	Not at all	509	50.2%
	Yes, a little	246	24.3%
	Yes, a lot or some	72	7%
	Don’t know	187	18.4%

^a^Percentages may not sum to 100% due to rounding.

## Discussion

The present study evaluated the effectiveness of a Web-based intervention to increase PA and improve health among physically inactive persons in a real-life setting. At follow-up we did not find any significant differences in PA and health measurements between the website and control groups. Nevertheless, participants in the website group increased their leisure-time PA and total PA in minutes/week according to active use of the intervention website.

One of the fundamental methodological problems in eHealth trials is that a proportion of people in the intervention group will not use the intervention or will use it only sparingly [35]. In this study, 7% logged on more than once and only 2% used the website as intended in the website group. With such a low use of the intervention website, a null finding in PA and health in the website group is not surprising. We recruited the participants from a large health examination survey. In the planning phase this seemed an ideal and easy way to enroll participants, but the percentage of nonusers in the study proved this wrong. Embedding the intervention in a large survey probably influenced the usage rate considerably. As stated, the main reason for nonuse in the intervention group was that participants simply forgot about the intervention. In a review of 15 Web-based PA interventions [22], the attrition rate ranged between 7% and 69% with an overall attrition of 27%, which is much lower than in the present study. A key feature of successful Web-based PA interventions is that studies enrolled self-selected volunteers [36-42]. It is likely that the participation of these highly motivated individuals resulted in higher retention and usage rates. Surprisingly, some participants in our study reported that they had logged on to the intervention website, even though reports showed that they never logged on. We believe that this was because the intervention was embedded in the survey, which could have led to confusion in some of the participants. The mean age was 50 years and the low computer skills among older participants might also be a reason for the above.

Other reasons that may explain the high number of nonusers in the study could be that Web-based interventions require more active participation than other types of mass media interventions, such as print material. To read the suggestions and use the website tools, participants have to sit at a computer and log on to the intervention. This kind of active participation requires time and effort from the participants, which may attract only highly motivated individuals [17]. If discontinuing the intervention, users are not obliged to explain why. In addition, a behavioral gap between positive intentions and behavior change has been demonstrated [43], which means that, despite 53% of the participants in this study intending to be more physically active, many did not act on this desire.

In this study we found an increase of leisure-time PA and total PA per week for the active users of the intervention. For these active and motivated participants, single-time feedback was sufficient to change PA level. The results are very much in line with a study by Smeets et al, who found that single-tailored feedback did not have an impact on the study population in a Dutch Web-based intervention [44]. However, when analyzing motivated users separately, Smeets and colleagues found an increase in PA level. Johnson and Wardle [37] examined the effect of a commercial Web-based weight loss program, using self-monitoring and feedback, on diet and PA and found that participants in the study lost a significant amount of weight. However, the study included only individuals who paid at least a month’s subscription and recorded weight spanning at least 28 days, supporting that self-engagement and true users can achieve a positive effect using a Web-based intervention.

A review of PA interventions found that studies with more supervision and contact through texting and email with participants were more successful and more often reported positive outcomes on PA than did studies with few contacts [22]. Research has shown that forming a new habit (ie, automaticity for the behavior) requires a large number of repetitions [45]. A single-time intervention may raise awareness and interest but, to progress further, additional intervention interactions are needed. An approach differing from the single-feedback and static website was seen in a successful Web-based tobacco cessation intervention [46]. There, the authors organized the program content into multiple pieces that were made available to the participants sequentially and for a restricted period. In this way, participants progressed through a predetermined sequence of modules with restricted freedom, referred to as tunneling. Furthermore, the program combined email, Web, interactive voice response, and texting, and for 54 weeks comprised more than 400 contacts. Because a change in behavior and increase in PA require great effort from unmotivated participants, it is possible that single-tailored feedback is insufficient to change behavior and is simply too brief an intervention. One Web-based PA intervention [47] examined the relationship between the individual and his or her environment and found a positive effect by including environmental information in the feedback, especially among overweight participants. This approach requires more detailed tailoring but should be considered in future interventions, as the outdoor environment and facilities nearby set the scene for PA. The results of this intervention show that the step from intention to action (ie, signing up to an intervention and using it in practice) should receive considerably more attention to improve the exposure or use of the intervention.

We did not find any improvements in health measurements at 3-month follow-up in the website group compared with the control group, not even when dividing participants into three subgroups according to their use of the intervention website. The possible increase in PA might not have been sufficient to improve health measurements in already-healthy adults if the increase was not in moderate to vigorous activity and for a considerable amount of time. Further, more than 3 months may be necessary to allow for physiological changes to occur [48].

Some methodological limitations must be considered in this study. The IPAQ was developed to estimate the PA of individuals in different domains. The validity of the IPAQ as a tool to measure changes in PA behavior may be questioned. Nonetheless, it has been used in several PA Web-based interventions [36,41,42,49-51]. Pedersen et al [52] also used the IPAQ in a workplace intervention study and could not detect a 1 hour/week change in PA at work, even though participants were being monitored and the PA 1 hour/week actually took place. Their use of the IPAQ may therefore have led to an underestimation of the effect of the intervention.

We found that active users of the intervention achieved a positive effect on PA. However, conclusions in randomized controlled trials should be drawn from consideration of differences between groups rather than in a subsample of the intervention group [53], since the validity of between-group comparisons established by randomization is not preserved in this analysis.

The high number of participants is a strength of this study, as subgroup analyses were possible. We found that the age groups 44–65 and 65+ years and motivated users were more likely to log on to the intervention website. Nevertheless, we found no change in PA in these groups despite their greater likelihood of logging on to the intervention.

We rate the external validity of this study high, as we used intention-to-treat analyses and recruited participants from a generalizable population, which enables assessment of the preventive potential if translated into practice.

The relatively high percentage of participants who were lost to follow-up is a limitation, although dropout was equally distributed between the website group and the control group and is thus not expected to influence the results.

### Conclusion

Web-based research is still in an early stage, with many lessons to be learned. The question is how we can move forward and develop interventions that can change PA behavior. The finding in this study suggests that active users of a Web-based intervention can achieve a positive effect. However, for unmotivated users, single-tailored feedback may be too brief. Future research should focus on the step from intention to action and on developing more sophisticated interventions. This is seen in Web-based smoking cession interventions, which combine different types of media and have many contacts, and thereby have the potential to reach both motivated and unmotivated sedentary individuals.

## Multimedia Appendix 1

Screen shoots recruitment of participants and intervention website.

## Multimedia Appendix 2

CONSORT E-Health Checklist V1.6.1 [54].

## Abbreviations

BMI: body mass index
DANHES: Danish Health Examination Survey 2007–2008
IPAQ: International physical activity questionnaire
PA: physical activity
