# Supplementary material for: Effect of a Web-Based Intervention to Promote Physical Activity and Improve Health Among Physically Inactive Adults: A Population-Based Randomized Controlled Trial
Source: J Med Internet Res. 2012 Oct 30;14(5):e145. doi: 10.2196/jmir.2109 (PMC3510714; doi:10.2196/jmir.2109)
Supplement: Supplementary file 2 [file jmir_v14i5e145_app2.pdf]

| CONSORT-EHEALTH Checklist V1.6 Report | Manuscript Number | 2109 |
| --- | --- | --- |
| Date completed | 8/25/2011 14:50:29 | |
| by | Andreas Wolff Hansen | |
| Effect of a web-based intervention to promote physical activity and improve health among physically inactive adults - A population-based randomized controlled trial | | |
| TITLE | | |
| **1a-i) Identify the mode of delivery in the title** | | |
| see below<br>"Effect of a web-based intervention to promote physical activity and improve health among physically inactive adults - A population-based randomized controlled trial" | | |
| **1a-ii) Non-web-based components or important co-interventions in title** | | |
| N/A | | |
| **1a-iii) Primary condition or target group in the title** | | |
| see below<br>"Effect of a web-based intervention to promote physical activity and improve health among physically inactive adults - A population-based randomized controlled trial" | | |
| ABSTRACT | | |
| **1b-i) Key features/functionalities/components of the intervention and comparator in the METHODS section of the ABSTRACT** | | |
| "The intervention website was founded on the theory of stages of change and planned behaviour and, apart from a forum page where a physiotherapist answered questions about PA and training, fully automated." | | |
| **1b-ii) Level of human involvement in the METHODS section of the ABSTRACT** | | |
| "The intervention website was founded on the theory of stages of change and planned behaviour and, apart from a forum page where a physiotherapist answered questions about PA and training, fully automated." | | |
| **1b-iii) Open vs. closed, web-based (self-assessment) vs. face-to-face assessments in the METHODS section of the ABSTRACT** | | |
| "Physically inactive adults (n=12,287) participating in a nationwide e-health survey and health examination in Denmark were randomly assigned to either intervention (website) (n=6,055) or a no-intervention control group (n=6,232) in 2008."<br>"A subgroup of participants (n=1,190) was invited to a follow-up health examination at 3 months."<br><br>Not possible to blind when the intervention is access to a web-site. | | |
| **1b-iv) RESULTS section in abstract must contain use data** | | |
| Less than 25% of the participants logged-on to the website once and only 7% logged on frequently. No difference in PA level was found between the website and the control group at 3 and 6 months' follow-up<br><br>Reported in methods of abstract.<br>"Physically inactive adults (n=12,287) participating in a nationwide e-health survey and health examination in Denmark were randomly assigned to either intervention (website) (n=6,055) or a no-intervention control group (n=6,232) in 2008."<br>"A subgroup of participants (n=1,190) was invited to a follow-up health examination at 3 months." | | |
| **1b-v) CONCLUSIONS/DISCUSSION in abstract for negative trials** | | |
| "Based on our findings, we suggest that active users of a web-based PA intervention can improve the level of PA. However, for unmotivated users, single-tailored feedback may be too brief. Future research should focus on developing more sophisticated interventions with potential to reach both motivated and unmotivated sedentary individuals." | | |
| INTRODUCTION | | |
| **2a-i) Problem and the type of system/solution** | | |

| | | |
|---|---|---|
| see below<br>"However, evidence supporting the one or the other is inconclusive [22;23]. Many web-based interventions conducted to date have limitations. Some studies use an inadequately powered sample, lack a no-information control group, have a large drop-out rate or rely only on self-reported outcome measures. " | | |
| **2a-ii) Scientific background, rationale: What is known about the (type of) system** | | |
| See below<br>"Web-based interventions have been successfully applied to achieve lifestyle improvement and health behaviour change targeting weight loss, stress management, fall-related injuries, smoking cessation and heavy drinking [9-12]. The Internet has several advantages in delivering health promotion, for example, cost- and time-effectiveness, 24 hour-accessibility, and generation of instant personalized or individually tailored feedback [13-15]. Individually tailored feedback, rather than a more general prevention message, is likely to be more effective as users can identify with relevant personal information rather than general information [15-19]. Overall, web-based interventions provide cost-time efficient means of delivering individually targeted lifestyle modification at a population level. Based on the existing web-based randomized controlled trials (RCT) in relation to PA, it is unclear whether the Internet can effectively deliver PA interventions [20-22]. A review from 2009 [23] identified web-based PA interventions in primary prevention; only four of 16 studies, had above average external validity and reported a positive, between-group effect in PA." | | |
| METHODS | | |
| **3a) CONSORT** | | |
| see below<br>"The aim of this study was to examine whether PA was increased among inactive persons in a large population with an automated web-based intervention. More specifically, to determine whether access to a website with individually tailored feedback on PA level and suggestions to increase PA resulted in improvements in self-reported PA, anthropometrics and physiological measurements in a intervention group compared with a no-information control group. " | | |
| **3b-i) Bug fixes, Downtimes, Content Changes** | | |
| "A technical error gave some participants in the control group access to the website and resulted in exclusion of 895 participants."<br><br>"Due to a technical error, only half the participants were invited to answer the 3 month follow-up questionnaire." | | |
| **4a-i) Computer / Internet literacy** | | |
| N/A since partipants were recruted from an e-health study. | | |
| **4a-ii) Open vs. closed, web-based vs. face-to-face assessments:** | | |
| "Participants who met the inclusion criteria were identified by a screening program and were invited to join the intervention study at the end of the questionnaire in DANHES. If willing to participate, each participant was randomly assigned by the registration program to either intervention (website) or a no-intervention control group. The only incentive given to participants was the possibility of being assigned to the intervention group. Blinding was not feasible.<br>The participants in the website group received an e-mail with a link to a PA website immediately after allocation to the website group. In addition, relevant data from the health survey were automatically transferred to the intervention website. To access the website the participants were required to log-on to the website, using the same personal username and password given in the health survey." | | |
| **4a-iii) Information giving during recruitment** | | |
| "Participants who met the inclusion criteria were identified by a screening program and were invited to join the intervention study at the end of the questionnaire in DANHES. If willing to participate, each participant was randomly assigned by the registration program to either intervention (website) or a no-intervention control group. The only incentive given to participants was the possibility of being assigned to the intervention group. Blinding was not feasible."<br>"All participants gave informed consent before being enrolled in the study. The study was approved by The National Committee on Biomedical Research Ethics in Denmark (H-D-2008-035)." | | |
| **4b-i) Report if outcomes were (self-)assessed through online questionnaires** | | |
| "After 3 months and after 6 months, all participants were invited by e-mail to answer a follow-up questionnaire. In addition, the follow-up questionnaire included questions about use of the website for the website group."<br><br>"From three selected municipalities in the intervention study, 1200 participants were invited to a follow-up health examination after 12 weeks. The participants were invited by e-mail, which was sent 3–4 weeks prior to the examination. If the participant did not respond, a reminder e-mail was sent one week after the first. The follow-up examination included the same measurements as the baseline health examination and the same test procedures were followed. The follow-up examination was blinded to the examiners." | | |

| | | |
|---|---|---|
| **4b-ii) Report how institutional affiliations are displayed** | | |
| "The DANHES was used to recruit participants and as baseline assessment. The intervention study was conducted in 11 of the 13 municipalities participating in DANHES during May 2008–May 2009. Two municipalities were not included in the intervention study as one served as a pilot study for DANHES and the second used paper questionnaires."<br><br>"The interventions was conducted by the same research group who conducted the DANHES." | | |
| **5-i) Mention names, credential, affiliations of the developers, sponsors, and owners** | | |
| N/A no company, affiliation sponsors were mentioned when invited to join the intervention study.<br>"All participants gave informed consent before being enrolled in the study. The study was approved by The National Committee on Biomedical Research Ethics in Denmark (H-D-2008-035)." | | |
| **5-ii) Describe the history/development process** | | |
| "The intervention website was founded on the theory of stages of change [25] and planned behaviour [26]. First, we identified determinants in the theory, which included: intentions, attitudes, self-efficacy, social support and knowledge. Second, key objectives were specified for each determinant and it was decided where in the intervention the objectives would be implemented (Table 1). The key objectives were used in the wider planning of the intervention to specify how objectives could be translated into action in the real-life intervention."<br>"The content of the website was developed by the research team. Two professional web companies did the graphical design and implementation. The intervention was pre-tested among experts and representatives of the target population. We tested screening of participants, invitation to the intervention, automatic generation of individually tailored advice, e-mail generation and the general usability of the website. Furthermore, the first municipality participating in the study (n=1,298) served as a pilot study, and comments and suggestions from the participants were used to fine tune the website. " | | |
| **5-iii) Revisions and updating** | | |
| Was not documented during development. | | |
| **5-iv) Quality assurance methods** | | |
| "The content of the website was developed by the research team. Two professional web companies did the graphical design and implementation. The intervention was pre-tested among experts and representatives of the target population. We tested screening of participants, invitation to the intervention, automatic generation of individually tailored advice, e-mail generation and the general usability of the website. Furthermore, the first municipality participating in the study (n=1,298) served as a pilot study, and comments and suggestions from the participants were used to fine tune the website. " | | |
| **5-v) Ensure replicability by publishing the source code, and/or providing screenshots/screen-capture video, and/or providing flowcharts of the algorithms used** | | |
| It has not been possible to have an online version of the website after the intervention.<br>But see Supplementary files 1 for screen shoots of the intervention website. | | |
| **5-vi) Digital preservation** | | |
| It has not been possible to have an online version of the website after the intervention.<br>But see Supplementary files 1 for screen shoots of the intervention website. | | |
| **5-vii) Access** | | |
| "The participants in the website group received an e-mail with a link to a PA website immediately after allocation to the website group. In addition, relevant data from the health survey were automatically transferred to the intervention website. To access the website the participants were required to log-on to the website, using the same personal username and password given in the health survey." | | |
| **5-viii) Mode of delivery, features/functionalities/components of the intervention and comparator, and the theoretical framework** | | |

| | | |
|---|---|---|
| "The intervention website was founded on the theory of stages of change [25] and planned behaviour [26]. First, we identified determinants in the theory, which included: intentions, attitudes, self-efficacy, social support and knowledge. Second, key objectives were specified for each determinant and it was decided where in the intervention the objectives would be implemented (Table 1). The key objectives were used in the wider planning of the intervention to specify how objectives could be translated into action in the real-life intervention.<br><br>The website was structured as three major parts: 1) a personal page, which included individually tailored PA advice and a personal profile, 2) a page with training programs and general recommendations, and 3) a forum/discussion page for questions from participants.<br>The individually tailored PA advice consisted of three parts: a general introduction; normative feedback, which related the participants' PA to the current PA recommendations; and general advice about using the different tools on the website. The normative feedback was based on the summarized PA time of participants' answers in the IPAQ. Feedback was given in the domains: everyday activity, fitness training, and strength training. In each domain we defined categories of which the participants received tailored feedback according to their level of PA. The categories were partly based on PA recommendations from the Danish National Board of Health translated into min/week [27] together with analyses from answers in the IPAQ from two municipalities. The analyses showed that the categories needed to be wide because of over reporting in the IPAQ. The categories were: everyday activity, al time from activity in the IPAQ summarized: low (<1200 min/week), moderate (1200–3500 min/week) and high (>3500 min/week); fitness training, summarized time from vigorous and moderate intensity activity from transport and the leisure time domain and moderate intensity from domestic domain in the IPAQ: low (<40 min/week), moderate (40–350 min/week) and high (>350 min/week); and strength training, summarized time from highest intensity level in the four domains in the IPAQ: low (<100 min/week), moderate-high (>100 min/week). Participants of 60+ years were given extra advice regarding the importance of strength training. With the purpose of monitoring their progress during the coming months, the participants could register personal data, such as waist circumference and the result of a short fitness and strength test in the personal profile. This kind of biofeedback was used to keep the participants motivated during the intervention. Furthermore, current activity per day could be calculated with a short activity calculator, and it was possible to set goals for the following four weeks. The four weeks for goal-setting was decided as a balance between time for seeing results and retaining motivation. All participants were encouraged to make a personal profile to set their goals, monitor progress and implement their goals.<br>The part of the website which included the page with the training programs and general recommendations was structured in the same way as the PA advice in the three domains: everyday activity, fitness training, and strength training. The participants were encouraged to go through the different suggestions and programs and pick the ones best suited to them based on the individually tailored advice and goals set by each participant. General information about motivation and relevant links were also present on the website.<br>On the forum/discussion page, a physiotherapist, with experience from counselling on PA, answered all questions about PA and training from participants. In addition, participants could share experiences and give each other tips or search for training partners in a second forum.<br>The tailored PA advice was kept short so as not to overload the participant with information, and apart from training programs and general recommendations, as a means of following the theory of stages of change [25]. The model describes how change is a process involving progress through a series of stages and includes the following stages: pre-contemplation, contemplation, preparation, action, maintenance and termination. Hence, participants ready for change and already in the action stage could make a personal profile, set goals and find training programs. Participants in the pre-contemplation to preparation stage who did not log-on to the website or not make a personal profile were sent two e-mail reminders to encourage them to become involved with the intervention. Participants in the maintenance stage who made a personal profile were sent reminders and encouraging e-mails to keep the profile updated after 4, 8, 12 and 16 weeks." | | |
| **5-ix) Describe use parameters** | | |
| "The participants were encouraged to go through the different suggestions and programs and pick the ones best suited to them based on the individually tailored advice and goals set by each participant. General information about motivation and relevant links were also present on the website. "<br>"All participants were encouraged to make a personal profile to set their goals, monitor progress and implement their goals." | | |
| **5-x) Clarify the level of human involvement** | | |
| "On the forum/discussion page, a physiotherapist, with experience from counselling on PA, answered all questions about PA and training from participants. In addition, participants could share experiences and give each other tips or search for training partners in a second forum." | | |
| **5-xi) Report any prompts/reminders used** | | |

| | | |
|---|---|---|
| "The participants in the website group received an e-mail with a link to a PA website immediately after allocation to the website group. In addition, relevant data from the health survey were automatically transferred to the intervention website. To access the website the participants were required to log-on to the website, using the same personal username and password given in the health survey."<br>"Participants in the pre-contemplation to preparation stage who did not log-on to the website or not make a personal profile were sent two e-mail reminders to encourage them to become involved with the intervention. Participants in the maintenance stage who made a personal profile were sent reminders and encouraging e-mails to keep the profile updated after 4, 8, 12 and 16 weeks. "<br>"From three selected municipalities in the intervention study, 1200 participants were invited to a follow-up health examination after 12 weeks. The participants were invited by e-mail, which was sent 3–4 weeks prior to the examination. If the participant did not respond, a reminder e-mail was sent one week after the first." | | |
| **5-xii) Describe any co-interventions (incl. training/support)** | | |
| N/A | | |
| **6a-i) Online questionnaires: describe if they were validated for online use and apply CHERRIES items to describe how the questionnaires were designed/deployed** | | |
| I have no knowledge if these questions have been validated for an online specifically. We have a manuscript in press about online use of IPAQ. | | |
| **6a-ii) Describe whether and how "use" (including intensity of use/dosage) was defined/measured/monitored** | | |
| ". In addition, the follow-up questionnaire included questions about use of the website for the website group. "<br>"Here we divided the participants of the website group into three groups according to user activity (no log-on, log-on once and log-on more than once)" | | |
| **6a-iii) Describe whether, how, and when qualitative feedback from participants was obtained** | | |
| N/A | | |
| **7a-i) Describe whether and how expected attrition was taken into account when calculating the sample size** | | |
| "We assumed that reasonable effect of the intervention on total time of physical activity estimated by the IPAQ would be around 12% and 5% for the intervention and control group, respectively. With a power of 80% probability of detecting a 12% vs. 5% difference as statistically significant at the 5% level we calculated the minimum sample size to be 250 in each group. We expected that approx. 50% of the participants in DANHES were sedentary. Assuming that 80% accepted participation and 25% were lost to follow-up, this would still give us a large population and hence ensuring sufficient power. " | | |
| **7b) CONSORT** | | |
| N/A | | |
| **8a) CONSORT** | | |
| "If willing to participate, each participant was randomly assigned by the registration program to either intervention (website) or control group" | | |
| **8b) CONSORT** | | |
| "If willing to participate, each participant was randomly assigned by the registration program to either intervention (website) or control group" | | |
| **9) CONSORT** | | |
| N/A | | |
| **10) CONSORT** | | |
| "If willing to participate, each participant was randomly assigned by the registration program to either intervention (website) or control group" | | |
| **11a-i) Specify who was blinded, and who wasn't** | | |
| For participants "Blinding was not feasible."<br>At follow-up health examination "The follow-up examination was blinded to the examiners. " | | |
| **11a-ii) Discuss e.g., whether participants knew which intervention was the "intervention of interest" and which one was the "comparator"** | | |
| N/A | | |
| **11b) CONSORT** | | |
| N/A | | |
| **12a) CONSORT** | | |

| | | |
|---|---|---|
| "Results were primarily analysed as intention to treat analyses with the use of last observation carried forward to account for missing data at follow-up.<br>Completer analyses were performed with only participants included who completed the follow-up health examination or questionnaire.<br>Website usage was assessed by the follow-up questionnaire and combined by information provided by the company who were responsible for the website who recorded if a participant logged on.<br>Odds ratios of being an active user (several log-ons and one/none) were calculated in relation to sex, highest education level (<10, 10–12, 13–14, 15+ years), age groups (18–44, 45–64, 65+ years) and motivation to be more active (yes, yes/maybe, no) by the use of logistic regression. " | | |
| **12a-i) Imputation techniques to deal with attrition / missing values** | | |
| "Results were primarily analysed as intention to treat analyses with the use of last observation carried forward to account for missing data at follow-up.<br>Completer analyses were performed with only participants included who completed the follow-up health examination or questionnaire." | | |
| **12b) CONSORT** | | |
| "As a post-hoc outcome measure, a secondary analysis was carried out among active users of the intervention. Here we divided the participants of the website group into three groups according to user activity (no log-on, log-on once and log-on more than once) and assessed level of physical activity. Furthermore we calculated odds ratios of being an active user of the website." | | |
| RESULTS | | |
| **13a) CONSORT** | | |
| See flow chart Figure 1. | | |
| **13b) CONSORT** | | |
| See flow chart Figure 1 | | |
| **13b-i) Attrition diagram** | | |
| See flow chart Figure 1 and figure 2 | | |
| **14a) CONSORT** | | |
| The intervention study was conducted in 11 of the 13 municipalities participating in DANHES during May 2008–May 2009. | | |
| **14a-i) Indicate if critical “secular events” fell into the study period** | | |
| We are not aware that the registration program or web-site had downtime during the intervention. | | |
| **14b) CONSORT** | | |
| N/A | | |
| **15) CONSORT** | | |
| see table 1 | | |
| **15-i) Report demographics associated with digital divide issues** | | |
| not shown | | |
| **16-i) Report multiple “denominators” and provide definitions** | | |
| Both ITT and completers analysis were performed. Furthermore active users were analyced. | | |
| **16-ii) Primary analysis should be intent-to-treat** | | |
| "Results were primarily analysed as intention to treat analyses with the use of last observation carried forward to account for missing data at follow-up. Completer analyses were performed with only participants included who completed the follow-up health examination or questionnaire." | | |
| **17a) CONSORT** | | |
| Yes see table 4+5 | | |
| **17a-i) Presentation of process outcomes such as metrics of use and intensity of use** | | |
| Yes "Here we divided the participants of the website group into three groups according to user activity (no log-on, log-on once and log-on more than once) "<br>Figure 2 | | |

| | | |
|---|---|---|
| **17b) CONSORT** | | |
| N/A | | |
| **18) CONSORT** | | |
| "As a post-hoc outcome measure, a secondary analysis was carried out among active users of the intervention. Here we divided the participants of the website group into three groups according to user activity (no log-on, log-on once and log-on more than once) and assessed level of physical activity. Furthermore we calculated odds ratios of being an active user of the website." | | |
| **18-i) Subgroup analysis of comparing only users** | | |
| "When the participants were divided into three groups according to use of the intervention website (no log-on, log-on once and log-on more than once), a significant difference was found between the groups in PA in leisure time and total PA (Figure 2). Dividing participants from the website group who participated in the follow-up health examination into the same three subgroups of website use did not show significant differences (data not shown)." | | |
| **19) CONSORT** | | |
| N/A | | |
| **19-i) Include privacy breaches, technical problems** | | |
| "Due to a technical error, only half the participants were invited to answer the 3 month follow-up questionnaire. "<br>"A technical error gave some participants in the control group access to the website and resulted in exclusion of 895 participants" | | |
| **19-ii) Include qualitative feedback from participants or observations from staff/researchers** | | |
| "Furthermore, the first municipality participating in the study (n=1,298) served as a pilot study, and comments and suggestions from the participants were used to fine tune the website." | | |
| DISCUSSION | | |
| **20-i) Typical limitations in ehealth trials** | | |
| "Some methodological limitations must be considered in this study. The IPAQ has been developed to estimate PA of individuals in different domains. The validity of the IPAQ as a tool to measure changes in PA behaviour may be questioned. Nonetheless, it has been used in several PA web-based interventions [36;41;42;49-51]. Pedersen et al. [52] also used the IPAQ in a workplace intervention study and could not detect a 1 hour/week change in PA at work, even though participants were being monitored and the 1 hour/week actually took place. The use of the IPAQ may therefore have led to an underestimation of the effect of the intervention.<br>We found that active users of the intervention could achieve a positive effect on PA. However, conclusions in RCTs should be drawn from consideration of differences between groups rather than in a subsample of the intervention group [53], since the validity of comparisons between groups established by randomization is not preserved in this analysis.<br>The high number of participants is a strength of this study as subgroup analyses were possible. We found that the age group of 44–65 and 65+ years and motivated users were more likely to log-on to the intervention website. Nevertheless, no change in PA was found in these groups despite the greater likeliness of logging-on to the intervention.<br>We rate the external validity of this study high as intention to treat analyses were used, and participants were recruited from a generalizable population which enables to assess the preventive potential if translated into practice.<br>The relative high percentage of participants who were lost to follow-up is a limitation. Though, the drop out was equally distributed among the website group and the control group and is thus not expected to influence the results. " | | |
| **21-i) Generalizability to other populations** | | |
| "We rate the external validity of this study high as intention to treat analyses were used, and participants were recruited from a generalizable population which enables to assess the preventive potential if translated into practice. " | | |
| **21-ii) Discuss if there were elements in the RCT that would be different in a routine application setting** | | |
| N/A | | |
| **22-i) Restate study questions and summarize the answers suggested by the data, starting with primary outcomes and process outcomes (use)** | | |

| | | |
|---|---|---|
| "The present study evaluated the effectiveness of a web-based intervention to increase PA and improve health among physically inactive persons in a real life setting. At follow-up we did not find any significant differences in PA and health measurements between the website and control group. " | | |
| **22-ii) Highlight unanswered new questions, suggest future research** | | |
| "The finding in this study suggests that active users of a web-based intervention can achieve a positive effect. However, for unmotivated users, single-tailored feedback may be too brief. Future research should focus on the step from intention to action and on developing more sophisticated interventions, as seen in web-based smoking cession interventions, which combine different types of media and have many contacts, and thereby have the potential to reach both motivated and unmotivated sedentary individuals. " | | |
| Other information | | |
| **23) CONSORT** | | |
| "Trial ID number: NCT01295203 (ClinicalTrials.gov)" | | |
| **24) CONSORT** | | |
| N/A Exist in danish language only | | |
| **25) CONSORT** | | |
| "Source of funding<br>This study was funded by TrygFonden, Denmark." | | |
| **X26-i) Comment on ethics committee approval** | | |
| "The study was approved by The National Committee on Biomedical Research Ethics in Denmark (H-D-2008-035)." | | |
| **x26-ii) Outline informed consent procedures** | | |
| "All participants gave informed consent before being enrolled in the study." | | |
| **X26-iii) Safety and security procedures** | | |
| N/A | | |
| **X27-i) State the relation of the study team towards the system being evaluated** | | |
| "Conflicts of interest<br>None to declare" | | |
